# An expert-informed multi-scale synthetic benchmark dataset for calendar-aware resource-constrained project scheduling (CA-RCPSP)

**DOI:** 10.1016/j.dib.2026.113256

**Published:** 2026-09-10

**Authors:** Md Aftab Uddin, Jaafar Gaber, Pierre Petitjean, François Cortinovis

**Affiliations:** a M-Plus Group, Lachapelle Sous Rougemont, 90360, France; b Université Marie et Louis Pasteur, UTBM, CNRS, Institut FEMTO-ST, Belfort, F-90010, France

**Keywords:** Project scheduling, Resource-constrained scheduling, Calendar constraints, Max-Plus algebra, Benchmark dataset, Expert elicitation, Disruption modelling, RCPSP, Synthetic data

## Abstract

We provide a synthetic, expertly designed benchmark dataset for Calendar-Aware Resource-Constrained Project Scheduling Problems (CA-RCPSP). The dataset consists of 12 problem instances across four scale levels: tiny (5–8 tasks), small (25–40 tasks), medium (120–180 tasks), and large (500–1000 tasks). It includes (i) a directed acyclic task graph (DAG) with six different task types, (ii) a heterogeneous resource pool consisting of CNC machines and skilled workers, (iii) a structured work calendar with definitions of shifts, lunch breaks and statutory holidays, and (iv) two to four stochastic disruption scenarios taken from a catalogue of fourteen disruption scenarios. The parameters in the scenarios were based on industrial conditions and were validated with two structured interviews performed with a senior production planning engineer, without violating confidentiality requirements of production records. All instances are created with fixed random seeds (base seed = 42 and scenario seed = 42+i) to ensure that the instances are byte-for-byte reproducible. Baseline results for three solvers that were used with the dataset are provided, but only as descriptive baselines to ensure that the dataset format can be used with different solver paradigms: a Max-Plus algebraic scheduler, Google OR-Tools CP-SAT, and Gurobi MIP. To our knowledge, the dataset is the first openly licensed, DOI-archived benchmark to distribute the work calendar as a structured first-class data object within each instance, and it addresses a structural gap in the widely used PSPLIB library [1], which omits calendar information. The dataset and the code to generate the data are released on Zenodo [2] under CC BY 4.0 at https://doi.org/10.5281/zenodo.20553817. The open source code repository can be found here: https://github.com/aftab-fr/carm-plus-dataset.

Specifications Table

**Table:** 

Subject	Industrial Engineering / Operations Research
Specific subject area	Resource-Constrained Project Scheduling; Calendar-Aware Scheduling; Disruption Management
Type of data	JSON scenario files; CSV solver-result files; Python generation and validation scripts
Data collection	Expert-informed stochastic generation. The parameters of the scenarios (task types, durations, resource costs, disruption rates, shift structures) were gathered from a senior production planning engineer through two structured interviews and then used to seed a pseudo random number generator (NumPy 1.26, base seed = 42). Three independent solver implementations were run on a single workstation to get solver results.
Data format	Raw (JSON, CSV); Analysed (validation summary JSON)
Parameters	Tasks n∈{5,8,25,40,120,150,180,500,750,1,000}; dependency insertion prob. ρ∈[0.15,0.30]; resources 5–40; shifts: standard-day / multi-shift / 24/7; disruption prob. p∈[0.0005,0.15]
Data source location	Not applicable (fully synthetic; no confidential production records used)
Data accessibility	Repository: Zenodo [2] https://doi.org/10.5281/zenodo.20553817 (CC BY 4.0). Code: GitHub https://github.com/aftab-fr/carm-plus-dataset
Related research article	None

## 1 Value of the Data

• PSPLIB [1] doesn’t contain any work-calendar information. Like PSPLIB, MMLIB [3] does not include calendar structures. In the literature, calendar-constrained extensions of the RCPSP can be found [4], and test instances for RCPSP/max-cal, adapted from existing RCPSP/max networks, are downloadable from the authors' institutional webpage [13]. In those test sets, however, the calendar enters as periods of resource unavailability, and shift structures, lunch breaks and statutory holidays are not represented as structured, semantically labelled objects, nor is any disruption information included; to the best of our knowledge, no openly licensed, DOI-archived benchmark distributes the work calendar as a first-class data object in this sense. This is filled by each instance in this dataset containing a full WorkCalendar object.
• The parameters were obtained through extensive discussions with a single experienced CNC manufacturing planner at one French facility, in order to capture the different aspects of practical operation, such as task-duration ranges, resource cost rates, disruption frequencies and shift structures. These were further cross-validated with the known manufacturing benchmark values found in the literature (see Experimental Design, Materials and Methods) further validating and giving reliability to the values obtained. A subset of the synthetic data was shown to the same expert to establish the usefulness of the data that was generated, and the expert verified that the data is representative of plausible scenarios that he has seen in industry. Consequently, the resulting dataset can be characterized as expert-informed synthetic rather than purely random: while the data generation process is algorithmic and reproducible, the underlying parameters are carefully constrained by industrial experience and domain knowledge. This approach ensures that the dataset balances realism and openness, making it suitable for rigorous testing and benchmarking of manufacturing planning algorithms. The single-expert, single-facility basis of these parameters, and the scope it places on the realism claim, are discussed in the Limitations section.
• The tiers are tiny, small, medium and large, allowing the researchers to test the algorithmic correctness on small problems and scalability up to 1000 tasks. There is no single existing dataset that currently covers this range.
• Every occurrence includes 2–4 disruption records from a fourteen-type disruption catalogue (machine failure, worker absence, cascade events etc.), allowing for robust and reactive scheduling methods to be evaluated. Reactive and robust scheduling research currently has few openly available benchmarks that ship disruption specifications alongside the base instance, so this catalogue is intended in particular for that community.
• Each scenario is seeded independently (seed = 42+i), so any instance can be regenerated in isolation without re-running the entire pipeline. For example, a researcher who needs to adjust a single medium instance can regenerate it directly from the random_seed value stored in its JSON file while leaving the other eleven files untouched.
• There are 3 solver comparisons in per_scenario_results.csv that give a quick descriptive baseline confirming that the data can be ingested by different solver paradigms; because the CP and MIP baselines reached the time limit on all large instances, cross-paradigm compatibility is fully demonstrated up to the medium scale. These JSON structures can be used to compare the performance of other algorithms such as genetic algorithms, column generation or any other CP/MIP model.
• The generation code is extensible: researchers can substitute parameter ranges elicited from experts in their own domain to produce domain-specific instances, and the CC BY 4.0 licence permits this reuse and redistribution without restriction.

## 2 Background

The RCPSP and its variants have been studied for decades, and shared instance libraries such as PSPLIB [1] and MMLIB [3] have long served as common ground for comparing scheduling algorithms. A simplifying assumption runs through most of these benchmarks: once a task starts, work proceeds without interruption. Shop-floor scheduling rarely behaves this way. Machines and operators follow fixed shifts, pause for lunch, and observe public holidays, so the time a task actually takes depends on where it falls in the working calendar. Calendar-constrained formulations of the RCPSP do exist [4], and the corresponding RCPSP/max-cal test instances are publicly available, yet those test sets represent the calendar as periods of resource unavailability rather than as a structured object (shifts, breaks, holidays) stored with each instance. That absence is what prompted us to assemble the dataset described here. The aim was to produce instances a scheduler can consume directly (a task graph, a mixed pool of CNC machines and workers, a work calendar, and a small set of disruptions) at sizes ranging from a few tasks up to a thousand. Parameter values were drawn from discussions with a senior production planner and from figures reported in the manufacturing literature, and each instance is regenerated from a fixed seed so that the files reproduce exactly.

## 3 Data Description

### 3.1 Repository layout

Listing 1.

**Listing 1:** Zenodo archive top-level structure.

### 3.2 Scenario inventory

Table 1 lists the twelve instances. Each instance is stored at data/scenarios/<scale>/<scenario_id>.json, so the Scenario ID column doubles as a file index. The dependency density column is the per-task predecessor-insertion probability ρ, not the graph edge-density ratio. The corresponding JSON field retains the name dependency_density for backward compatibility with the released files, but it stores this insertion probability and not a realised graph density; the two diverge noticeably at small n. The resulting edge density ($\frac{\left|E\right|}{\left(\begin{array}{c} n\\ 2 \end{array}\right)}$) is much smaller at large scales (≈ 0.001 for n=1,000), which is consistent with the sparse precedence structures observed in real industrial projects [5].

**Table 1:** The twelve CA-RCPSP benchmark instances (ρ = per-task dependency insertion probability; Res. = number of resources; Disrupt. = number of disruption records per instance). Dependency counts at the tiny scale fall below the unconstrained expectation for ρ = 0.30 as a consequence of acyclicity enforcement and transitive-edge pruning; see the discussion under Stage 1 in the Experimental Design, Materials and Methods section.

Scale	Scenario ID	Tasks	Deps	Res.	ρ	Disrupt.	Shift
Tiny	tiny_manufacturing_demo	6	1	5	0.30	2	Std. day
Tiny	tiny_assembly_demo	8	1	5	0.30	2	Std. day
Tiny	tiny_quality_demo	5	2	5	0.30	2	Std. day
Small	small_disrupted_manufacturing_25	25	9	10	0.25	4	Std. day
Small	small_multi_resource_35	35	26	10	0.25	2	Std. day
Small	small_shift_operations_40	40	20	10	0.25	2	Multi
Medium	medium_complex_manufacturing_120	120	50	20	0.20	3	Std. day
Medium	medium_multi_shift_factory_150	150	67	20	0.20	2	Multi
Medium	medium_supply_chain_180	180	70	20	0.20	3	Std. day
Large	large_industrial_scale_500	500	122	40	0.15	4	Multi
Large	large_enterprise_manufacturing_750	750	212	40	0.15	4	Multi
Large	large_mega_scale_factory_1000	1000	291	40	0.15	4	24/7

### 3.3 From expert narrative to DAG: A worked example

**Fig. 1:**
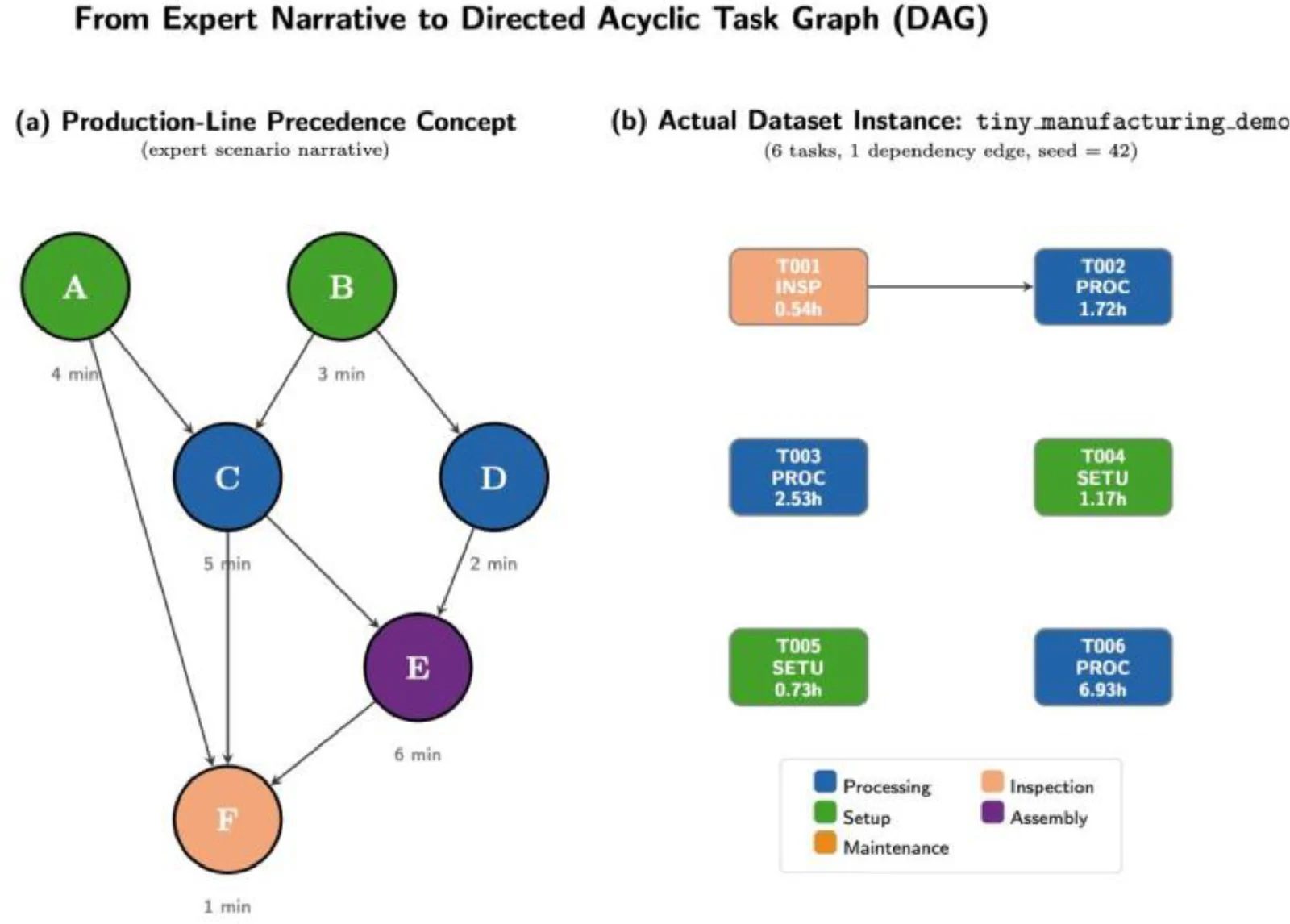
(a) Production-line precedence concept as collected from the domain expert; (b) corresponding actual dataset instance tiny_manufacturing_demo (*n* = 6 tasks, 1 dependency, seed = 42). Node colours identify task type (see legend).

Fig. 1 illustrates the conceptual bridge between an expert scenario description and the dataset’s internal representation. Panel (a) shows a stylised production-line precedence structure as an expert might describe it: six machines (A–F) with cycle times and precedence arrows indicating which machines must complete before others can begin. This type of workflow narrative, collected during structured interviews, constrains the generation parameters (task types, duration ranges, dependency density ρ) used in Stage 1. Panel (b) shows the corresponding actual instance (tiny_manufacturing_demo, n=6, seed = 42) as generated from those constraints. Each node encodes task type and duration; the single directed edge encodes the only precedence dependency present in this instance given ρ=0.30 and n=6.

### 3.4 JSON instance schema

Each scenario file is a self-contained JSON object. Listing 2 shows the abridged structure of tiny_manufacturing_demo.

**Listing 2:** Abridged JSON schema for tiny_manufacturing_demo (6 tasks, seed = 42). Two of the six tasks are shown; the remaining tasks follow the same schema. Comments are for illustration only; the deposited files contain no JSON comments.

**Field reference.**

• task_type: one of {processing, setup, transport, inspection, assembly, maintenance}.
• duration: task duration in decimal hours.
• required_skills: subset of the worker skill catalogue; every skill is guaranteed to be held by at least one resource.
• priority: integer 1 (low) to 5 (high).
• dependencies: list of [predecessor, successor] task-ID pairs encoding the precedence DAG.
• resource_type: Machine | Worker | Equipment.
• skills: proficiency map, values∈(0,1].
• shift_type: standard_day (08:00–17:00, Mon–Fri) | multi_shift (+ evening 17:00–01:00) | 24_7 (three 8 h shifts, all days).
• disruption.severity: low | medium | high | critical.
• disruption.probability: occurrence probability per scheduling horizon.
• expected_performance: indicative reference values (makespan_hours, resource_utilization) recorded in the scenario specification when each instance was designed, informed by the expert elicitation. They are not computed by any solver, are neither bounds nor ground truth, and should not be used to score algorithms; solver-computed makespans are reported in per_scenario_results.csv.

### 3.5 Result files

**Per-scenario solver comparison.** Table 2 reproduces data/results/per_scenario_results.csv. Blank cells indicate solver timeout (3600 s limit). Calendar compliance is the fraction of scheduled task intervals that fall strictly within working windows (shifts, excluding lunch breaks and public holidays).

**Table 2:** Per-scenario solver results (12 instances × 3 solvers). — = timeout. Bold marks the per-scenario extreme of each metric for readability; no solver ranking is implied.

Scenario	Solver	Task MK (h)	Solve (s)	Cal. compl.	Util.
*tiny_manufacturing*	Max-Plus	**1.84**	**0.002**	**1.000**	**0.782**
	OR-Tools	2.73	0.016	0.889	0.560
	Gurobi	2.31	0.037	0.944	0.547
*tiny_assembly*	Max-Plus	**2.62**	**0.002**	**1.000**	**0.777**
	OR-Tools	3.86	0.016	0.895	0.553
	Gurobi	3.35	0.050	0.953	0.546
*tiny_quality*	Max-Plus	**1.42**	**0.002**	**1.000**	**0.766**
	OR-Tools	2.29	0.017	0.898	0.564
	Gurobi	1.89	0.043	0.946	0.553
*small_25*	Max-Plus	**3.05**	0.120	**1.000**	**0.687**
	OR-Tools	4.90	0.008	0.919	0.604
	Gurobi	3.74	**0.001**	0.870	0.617
*small_35*	Max-Plus	**2.57**	0.189	**1.000**	**0.685**
	OR-Tools	3.88	0.008	0.914	0.597
	Gurobi	3.41	**0.001**	0.901	0.614
*small_40*	Max-Plus	**2.52**	0.190	**1.000**	**0.675**
	OR-Tools	4.06	0.007	0.920	0.595
	Gurobi	3.45	**0.001**	0.894	0.629
*medium_120*	Max-Plus	**2.58**	3.984	**1.000**	**0.703**
	OR-Tools	4.12	0.039	0.844	0.657
	Gurobi	3.30	**0.015**	0.842	0.676
*medium_150*	Max-Plus	**3.00**	5.578	**1.000**	**0.698**
	OR-Tools	4.60	0.047	0.869	0.670
	Gurobi	4.04	**0.015**	0.878	0.692
*medium_180*	Max-Plus	**2.86**	6.088	**1.000**	**0.705**
	OR-Tools	4.30	0.051	0.837	0.683
	Gurobi	3.59	**0.014**	0.855	0.679
*large_500*	Max-Plus	**2.73**	**694.7**	**1.000**	**0.731**
	OR-Tools	—	—	—	—
	Gurobi	—	—	—	—
*large_750*	Max-Plus	**3.06**	**1094.0**	**1.000**	**0.737**
	OR-Tools	—	—	—	—
	Gurobi	—	—	—	—
*large_1000*	Max-Plus	**3.84**	**1342.2**	**1.000**	**0.750**
	OR-Tools	—	—	—	—
	Gurobi	—	—	—	—

Note: this table is a solver ingestion compatibility check, not a comparative performance benchmark. Max-Plus attains a calendar compliance of 1.000 by algebraic construction, while the OR-Tools and Gurobi baselines use deliberately relaxed formulations that treat calendar windows as soft constraints, so their lower Task MK values are artificially optimistic (see accompanying text). The Task MK (h) column reports the per-task makespan, an ingestion metric (mean scheduled task duration under the instance calendar, stored as makespan_hours in per_scenario_results.csv), used here to confirm that each solver paradigm ingests and processes the calendar-annotated JSON correctly; it is not the full-project completion time and is not comparable to the end-to-end New MK (h) in Table 3.

**Fig. 2:**
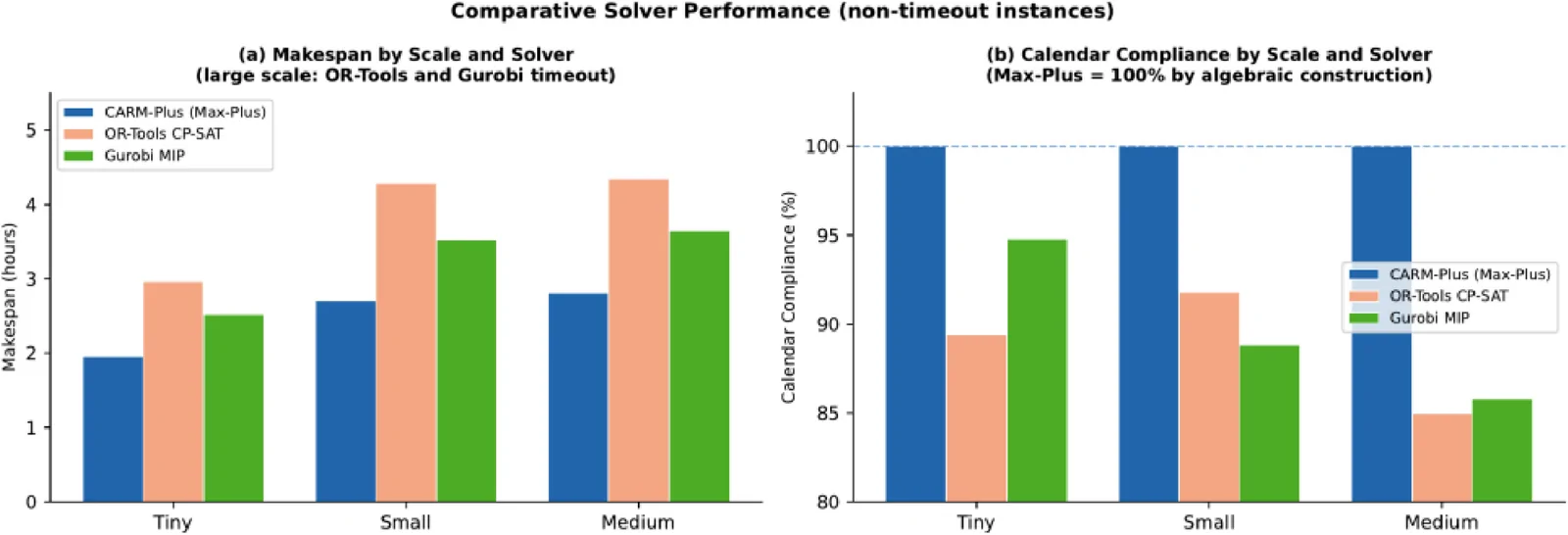
Descriptive baseline metrics on non-timeout instances (tiny, small, medium). (a) Task MK in hours (the per-task makespan; the makespan label on the panel axis refers to this metric); (b) calendar compliance rate. Max-Plus attains a compliance of 1.000 by algebraic construction, while the CP-SAT and MIP baselines use relaxed calendar formulations; the panels therefore verify solver ingestion rather than compare performance.

The results presented in Table 2 and Figure constitute a solver ingestion compatibility test rather than a comparative performance benchmark; they serve strictly as descriptive baselines meant to verify that the dataset can be successfully ingested by distinct solver paradigms (Algebraic, Constraint Programming, and Mixed-Integer Linear Programming). Blank cells in the results indicate that the solver reached a timeout under a standard 3600-second execution wall-clock limit. The sub-100% calendar compliance rates reported for standard CP-SAT and MIP paradigms stem from an intentional relaxation in the baseline model formulations, which do not natively treat the discrete, non-contiguous calendar arrays as hard constraints; the Max-Plus scheduler, by contrast, embeds the cumulative work-time function W(t) directly in its transition matrices and satisfies the calendar by construction. Consequently, their reported Task MK values represent an artificially optimistic lower bound, highlighting the modelling challenges that this dataset is designed to help the community resolve. No ranking of the three solvers should be inferred from Table 2 or Fig. 2. No calendar-exact CP or MIP encoding was developed for this release; constructing such encodings, and solving the large instances within practical time limits, remain open challenges that the dataset is intended to support.

**Disruption response.** Table 3 reproduces data/results/disruption_response.csv. Tests were conducted on the three medium-scale instances using the CARM-Plus reactive rescheduler. This experiment is separate from the solver runs reported in Table 2. Each disruption was injected into a baseline schedule with a pre-disruption makespan of 28.5 h (recoverable from any row of Table 3 as New MK divided by 1 + ΔMK/100), and the New MK column reports the makespan of the repaired schedule. The two tables report different-scope metrics and are not directly comparable: Task MK (h) in Table 2 is the per-task makespan, an ingestion check (mean scheduled task duration, used to verify calendar-aware processing across solver paradigms), whereas New MK (h) in Table 3 is the full end-to-end completion time of a complete multi-task, multi-resource instance rescheduled by CARM-Plus over the entire calendar horizon from the MK0 = 28.5 h baseline. No disruption-response experiments were conducted at the tiny, small or large scales. The medium tier was selected because its task counts and resource pools correspond most closely to the production setting from which the generation parameters were elicited, making it the most representative scale at which to demonstrate the reactive rescheduler. It is also the largest tier at which all three baseline solvers complete within the 3600 s limit. Extending the experiment to the remaining tiers is left to future work.

**Table 3:** CARM-Plus disruption response (medium-scale instances). Robustness (%) = $100\;\times \;(1\;-\;\frac{\Delta \text{MK}}{MK_{0}})$. MK0 = 28.5 h is the pre-disruption baseline makespan, identical for all three experiments.

Disruption type	Resp. (s)	New MK (h)	ΔMK (%)	Robustness (%)
Machine failure	<0.001	30.71	7.75	92.2
Worker absence	<0.001	37.57	31.81	68.2
Multiple cascade	<0.001	39.03	36.95	63.1
**Average**	**<0.001**	**35.77**	**25.50**	**74.5**

## 4 Experimental Design, Materials and Methods

### 4.1 Expert elicitation and parameter grounding

Scenario parameters were elicited through two structured interviews with a senior production planning engineer employed by M-Plus Group, the industrial partner in the CIFRE arrangement under which this work was carried out and an affiliation of three of the authors. The expert provided:

1. Typical task workflow structures for five production families (turning, milling, assembly, quality control, maintenance).
2. Shift configurations and statutory holiday calendars in operational use at the facility.
3. Historically observed disruption types, approximate per-shift occurrence frequencies, and typical durations.
4. Realistic counts of CNC machines and skilled workers, and indicative hourly cost ranges.

Both interview sessions followed a prepared question list covering the four topics above. Where the expert provided a range rather than a point value, the full range was retained as the sampling interval; where he expressed uncertainty, the range was widened to cover the corresponding published figures. The guide itself is not deposited, because its content reflects internal operational detail of the collaborating company and falls under the data confidentiality policy under which this work was carried out, the same constraint that led the dataset to be synthesised rather than drawn from production records; the elicitation stage therefore cannot be repeated verbatim by an independent researcher. Its outputs are nevertheless documented in full: the four topic areas above delimit what was asked, and every parameter range obtained from the sessions is reported in Table 4 alongside the published figure against which it was corroborated. The consequence for reproducibility is noted among the limitations. The expert's input was used to define the stochastic generation ranges shown in Table 4; no actual production schedules, job-order data, or personnel records were obtained or used. A sample of three generated instances (one per scale: tiny, small, medium) was subsequently reviewed by the same engineer, who confirmed that the task structures, resource configurations, and disruption scenarios were consistent with production conditions observed at the facility. The large instances (500 to 1000 tasks) were not part of this review, so their realism rests on the validated parameter ranges rather than on direct expert confirmation; this is noted in the Limitations section.

**Table 4:** Generation parameters and their empirical basis. ILO [6]; OEE [7]; CNC costs [8].

Parameter	Range	Basis
CNC machine cost	$30–$80/h	Expert; corroborated by CNC machining cost-structure analysis [8]
Worker cost	$15–35/h	Expert; W. European mfg. labour rates [6]
Machine efficiency η	0.80–1.00	Expert; OEE world-class benchmark ≈ 85% [7]
Processing duration	1–8 h	Expert (CNC job-lot cycle times)
Setup duration	0.5–2 h	Expert; SMED literature [9]
Machine failure prob.	0.01–0.15	Expert; OEE failure-rate data [7]
Dep. insertion prob. ρ	0.15–0.30	Expert; comparable to PSPLIB range [1]
Shift structure	08:00–17:00 Mon–Fri	Expert; EU Working Time Directive [10]
Annual statutory holidays	4 dates/year	Expert (French manufacturing calendar)

### 4.2 Generation pipeline

Figure summarises the four-stage pipeline implemented in code/generate_all_scenarios.py.

**Stage 1 — Task DAG.** N tasks are sampled from a weighted multinomial distribution over six types: processing (30%), setup (15%), transport (10%), inspection (15%), assembly (20%), maintenance (10%). Duration $d_{i}$ is drawn from a type-specific uniform distribution (e.g. $d_{i}\;∼\;U\left(1,\;8\right)$ h for processing). Precedence edges are inserted in topological index order: task j receives predecessor i<j where the dependency insertion probability is ρ∈{0.30,0.25,0.20,0.15} (for Tiny / Small / Medium / Large). An additional $\left\lfloor \frac{N\rho }{2}\right\rfloor$ random forward edges are inserted while maintaining acyclicity. Note that for the smallest instances (e.g., tiny_manufacturing_demo where n=6 and ρ=0.30), the strict application of topological constraints and edge-pruning to prevent redundant transitive paths can result in an actual dependency count (Deps = 1) lower than the statistical expected value of the unconstrained base density. This undershoot is systematic at the tiny scale: both tiny_manufacturing_demo (n = 6) and tiny_assembly_demo (n = 8) contain a single dependency edge each. The tiny instances are therefore suited to format ingestion and calendar-handling checks rather than to testing precedence-constraint behaviour, for which the small, medium and large scales provide denser precedence graphs.

**Stage 2 — Resource Pool.** Machines (CNC lathes, mills, drill presses, grinders, saws) and workers are instantiated proportional to scale: 2 machines + 3 workers (Tiny) to 15 machines + 25 workers (Large). A post-generation coverage guarantee ensures that every skill required by any task is held by at least one worker, preventing unassignable tasks. After the worker pool is sampled, any skill in the worker skill catalogue that remains uncovered is added to the skill map of the last generated worker at a fixed proficiency of 0.70. This repair step is deterministic and consumes no random draws, so it does not perturb the RNG stream on which reproducibility depends. Costs and efficiencies are drawn from the expert-grounded ranges in Table 4.

**Stage 3 — Work Calendar.** A WorkCalendar object is built via add_shift() and add_holiday() calls using one of three templates: standard-day (08:00–17:00, Mon–Fri, lunch 12:00–13:00); multi-shift (adds evening shift 17:00–01:00); 24/7 (three 8-hour shifts on all days). Figure illustrates the calendar structure and the cumulative work-time function W(t) used by the Max-Plus solver.

**Fig. 3:**
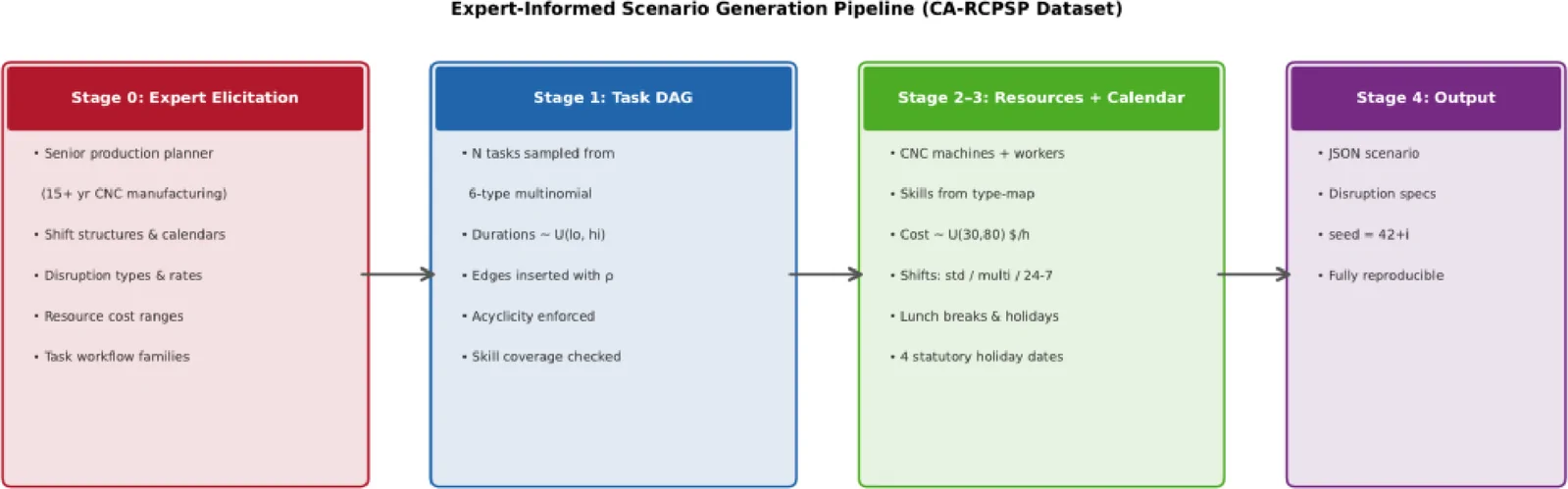
Four-stage expert-informed scenario generation pipeline. Stage 0 (expert elicitation) constrains the stochastic ranges used in Stages 1–4.

**Fig. 4:**
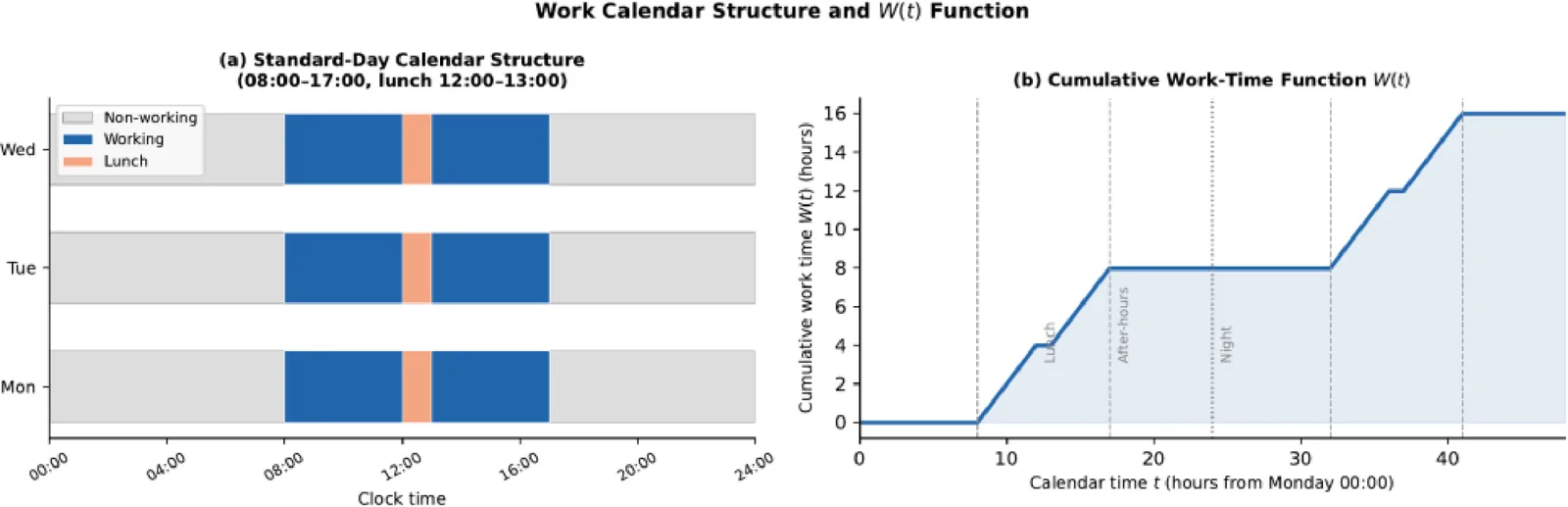
(a) Standard-day calendar showing working and non-working periods; (b) cumulative work-time function W(t) (flat segments correspond to non-working periods). CARM-Plus embeds W(t) directly into the (max,+) transition matrices.

**Stage 4 — Disruption Specifications.** Each instance receives 2–4 disruption records drawn from the fourteen-type catalogue in Table 5. The records are not sampled at random: they are assigned in the scenario specification to match each instance's operational theme (for example, medium_supply_chain_180 carries two delivery_delay records and one quality_rejection record), with severities, durations and occurrence probabilities set within the expert-elicited ranges. All disruption types used in any instance are members of this catalogue; the generation code raises a ValueError at runtime if a non-canonical type is encountered (Fig. 3, Fig. 4).

**Table 5:** Canonical disruption type catalogue. All types are drawn from this table; non-canonical types are rejected at generation time.

Type	Severity range	Typical duration
machine_failure	low – critical	1.5 – 24 h
worker_absence	medium – high	4 – 24 h
quality_issue	medium	2 h
supply_shortage	high	6 – 48 h
shift_change_delay	low – medium	1 – 2 h
resource_unavailable	high	8 h
calendar_change	medium	2 h
system_wide_failure	critical	24 h
delivery_delay	high	48 h
quality_rejection	medium	12 h
plant_shutdown	critical	48 h
worker_strike	critical	72 h
catastrophic_failure	critical	120 h
regulatory_shutdown	critical	96 h

### 4.3 Reproducibility

Each scenario is seeded independently before any stochastic sampling:  where scenario_index∈{0,…,11}. The actual seed is stored in each JSON file as random_seed. This design means each instance is individually reproducible and that inserting or reordering scenarios does not alter the RNG stream of any other instance.

### 4.4 Solver execution environment

To establish baseline execution performance and ensure reproducible benchmarking, all computational experiments were executed on a single workstation configuration detailed in Table 6. A maximum wall-clock timeout limit of 3600 s was strictly enforced across all problem scales to evaluate algorithmic scalability under realistic operational planning constraints.

**Table 6:** Hardware and software configuration.

Component	Configuration
CPU	13th Gen Intel Core i7–1365U
RAM	16.0 GB
OS	Microsoft Windows 11 Education
Python	3.10
OR-Tools	9.15.6755 (CP-SAT back-end) [11]
Gurobi	13.0.2 (LP/MIP) [12]
NumPy	1.26
Pandas	2.1
Base seed	42
Time limit	3600 s (all scales)

### 4.5 Dataset integrity validation

The script code/validate_dataset.py performs nine automated checks on every JSON instance:

1. All required top-level keys present.
2. Task IDs are unique.
3. Dependency edges reference valid task IDs.
4. DAG is acyclic (depth-first topological sort).
5. Resources have required fields and unique IDs.
6. Calendar has at least one shift defined.
7. Disruption records have required fields.
8. All disruption types are members of the canonical catalogue (Table 5).
9. Every task-required skill is held by at least one resource (no unassignable tasks).

All twelve instances pass all nine checks.

### 4.6 Reproduction quickstart

Listing 3.

**Listing 3:** Reproducing all 12 instances from scratch.

### 4.7 Minimal usage example

Listing 4.

**Listing 4:** Loading a deposited instance and reading its precedence, calendar and disruption fields.

The archive is versioned on Zenodo. The DOI cited in this article resolves to the release described here, while the Zenodo concept DOI for the record always resolves to the most recent version; the version number and release date of each deposit are recorded in CITATION.cff.

## Limitations

The dataset has several limitations that users should take into account. First, the generation parameters are grounded in the judgement of a single senior production planning engineer at one French CNC manufacturing facility. The elicited ranges were corroborated against published labour-cost, OEE and SMED figures (Table 4), but values typical of one facility may not transfer to aerospace, automotive, electronics or non-European manufacturing contexts, and the realism claims made in this article should be read within that scope. The interview guide used during elicitation is not deposited, because its content falls under the data confidentiality policy under which this work was carried out, so the elicitation stage cannot be replicated verbatim; the parameter ranges it produced are nevertheless reported in full in Table 4, each with its corroborating published source. Second, the work calendars encode French statutory holidays and European shift conventions only; instances for other calendar regimes must be regenerated with modified WorkCalendar templates. Third, the data are synthetic: the generator reproduces the structure of industrial scheduling problems but not the measurement noise, recording errors and undocumented shop-floor practices found in real production records. Fourth, the release contains twelve instances, which is adequate for format validation and descriptive baselines but modest relative to the combinatorial diversity of real scheduling problems; the open generation code mitigates this by allowing further instances to be produced. Fifth, the expert review covered one generated instance each at the tiny, small and medium scales, so the realism of the large instances (500 to 1000 tasks) is extrapolated from the validated parameter ranges rather than directly confirmed. Sixth, the CP-SAT and MIP baselines reached the 3600 s time limit on all large instances, so cross-paradigm solver compatibility is fully demonstrated only up to the medium scale. Finally, acyclicity enforcement and transitive-edge pruning cause the realised dependency counts of the tiny instances to fall below the nominal insertion probability ρ = 0.30, so these instances are best used for format ingestion and calendar-handling checks rather than for testing precedence-constraint behaviour. A further simplification concerns the duration distributions: task durations are drawn from type-specific uniform distributions, whereas processing times observed in production are frequently skewed or heavy-tailed. Substituting empirical or log-normal distributions in the generation code is a straightforward extension that we regard as the most useful next step for a future release.

## Ethics Statement

Not applicable. This article describes a fully synthetic, expert-informed dataset generated by computer code. No human subjects, animals, patient data, or confidential industrial records were used. The domain expert participated voluntarily in two structured interviews. Before the sessions he was informed that the discussions were being held to collect data for this research, and the purpose of the work and the intended uses of the elicited parameter ranges were explained to him. No personal data, identifying details or employment records were collected or stored, and the expert is not named in this article or in the deposited files.

## Declaration of generative AI And AI-assisted technologies in the writing process

During the preparation of this work the authors used Gemini in order to improve language clarity and readability. After using this tool, the authors reviewed and edited the content as needed and take full responsibility for the content of the publication.

## CRediT authorship contribution statement

**Md Aftab Uddin:** Conceptualization, Methodology, Software, Formal analysis, Investigation, Data curation, Writing – original draft, Visualization. **Jaafar Gaber:** Conceptualization, Methodology, Validation, Supervision, Writing – review & editing. **Pierre Petitjean:** Conceptualization, Resources, Supervision. **François Cortinovis:** Conceptualization, Resources, Supervision, Project administration.
